# Metabolomic signatures of colonic infection by *Brachyspira hyodysenteriae*

**DOI:** 10.1186/s13567-026-01727-9

**Published:** 2026-05-19

**Authors:** Lucía Pérez-Pérez, Cristina Galisteo, Laura de los Santos Castillo-Peinado, Sonia Tomé-Rodríguez, Feliciano Priego-Capote, Ana Carvajal, Héctor Arguello

**Affiliations:** 1 Departamento de Sanidad Animal, Facultad de Veterinaria, Universidad de León, León, Spain; 2 INDEGSAL, Universidad de León, León, Spain; 3 Departamento de Química Analítica, Facultad de Ciencias, Campus de Rabanales, Universidad de Córdoba, Córdoba, Spain; 4 Chemical Institute for Energy and Environment (IQUEMA), Campus de Rabanales, Universidad de Córdoba, Córdoba, Spain

**Keywords:** Swine dysentery, metabolome, pig, darrhea

## Abstract

Despite swine dysentery’s relevance in the pork industry, there are still gaps in our understanding of its pathogenesis and the impact of the infection in the gut. This study aimed to characterize the in vivo colonic metabolome of pigs experimentally infected with *Brachyspira hyodysenteriae* at the onset of fecal shedding (Early_inf group, *n* = 6) and during acute clinical disease characterized by mucohemorrhagic diarrhea (Acute_inf group, *n* = 8) compared with non-infected controls (*n* = 16). The metabolic profile of the colonic contents changed progressively with disease severity, showing an intermediate pattern in the Early_inf group between the control and the Acute_inf groups (*p* < 0.05). In acute disease, the metabolome was defined by increased concentrations of amino acids, carnitine derivatives, arachidic acid, 1,2-butanediol, and lactic acid, along with decreased levels of anti-inflammatory compounds. In the Early_inf group, increases were observed in amino acids, organic acids, amines, myo-inositol, quinoline, and 1,2-butanediol, whereas linolenic acid and oxalic acid decreased. Integrated analysis of the colonic metabolome and metagenome revealed a strong correlation between metabolic and microbial profiles, particularly in the Acute_inf group, where differential metabolites were associated with *B. hyodysenteriae*, *Campylobacter hyointestinalis*, and *Velocimicrobium ethanolgignens*. Metabolites showed high predictive potential for the disease stage, with lactic acid and arachidic acid being key markers of acute infection and dihydroxyacetone and leucine distinguishing early infection. Overall, this study reveals significant alterations in the colonic metabolome and its association with the microbiota during swine dysentery, providing new insights into the pathophysiology of the disease and contributing to the development of improved prevention and treatment strategies.

**Supplementary Information:**

The online version contains supplementary material available at 10.1186/s13567-026-01727-9.

## Introduction

Swine dysentery (SD) is an important enteric disease of pigs caused by strongly beta-hemolytic spirochetes, primarily *Brachyspira hyodysenteriae* and, more recently, *Brachyspira hampsonii* and *Brachyspira suanatina* [1]. The disease occurs in most countries with intensive pig production and is associated with substantial economic losses owing to impaired growth performance, high morbidity, and treatment and control costs [2]. Swine dysentery is characterized by severe colitis with mucohemorrhagic diarrhea in growing and finishing pigs [3]. The development of effective alternatives for disease prevention and control, which currently rely almost exclusively on biosecurity measures and antibiotic therapy [3, 4], demands a better understanding of the pathogenesis. *Brachyspira hyodysenteriae* induces mucosal ulceration, severe inflammation, and luminal hemorrhage [5, 6]. This pathological alteration of the large intestine impacts the microbiota composition as well [7, 8].

The metabolome comprises the metabolites produced as end products of intestinal and microbial metabolism. Its study is of interest in physiological and pathological processes [9, 10]. The development of omics disciplines has advanced the understanding of multiple diseases by enabling comprehensive and multifaceted analyses of biological systems [11]. In this context, metabolomics, focusing on the characterization of metabolites, provides a direct snapshot of the physiological and metabolic status of organisms [12, 13]. Therefore, metabolomics can be applied to identify predictive biomarkers and is increasingly used as a framework to understand the pathophysiological mechanisms underlying a wide range of diseases [14, 15].

Previous studies have reported alterations in carbohydrate metabolism in the plasma of pigs with mucohemorrhagic diarrhea induced by *B. hyodysenteriae*, including elevated glucose and lactate concentrations [16]. Exposure of porcine colon explants to this bacterium has shown that alanine, aspartate, and glutamate metabolism pathways; ketone body synthesis and degradation; and pyruvate metabolism are altered, with an accumulation of citrulline observed in the infected tissue [4]. In previous studies, we have characterized the alterations that *B. hyodysenteriae* prompts in the colon mucosa and colonic microbiota at different stages of SD [6, 7]. The objective of this study was to characterize the in vivo changes in the porcine colonic metabolome during the early and acute stages of *B. hyodysenteriae* infection to complete the understanding of the pathogenesis of the disease.

## Materials and methods

### *Brachyspira hyodysenteriae* challenge and sample collection

The experimental infection protocol was approved by the Animal Care and Supply Committee of the University of León (OEBA-ULE-010-2020), and the study design has been previously described in Pérez-Pérez et al. [6]. Briefly, 32 female, 7-week-old, crossbred pigs (Landrace × Large-White × Pietrain) from an SD-free farm were randomly allocated into two groups (*n* = 16 each) and housed in mirror-image pens within the biocontainment facilities of the University of León (Spain). Animals in the challenged group were orally inoculated with 30 mL of a broth culture of *B. hyodysenteriae* strain B‑204 (ATCC 31212) containing 5 × 10^8^ bacteria/mL. Within this group, half of the pigs (*n* = 8; Early_inf group) were euthanized 24 h after the first *B. hyodysenteriae*-positive fecal quantitative polymerase chain reaction (qPCR) result, which occurred at a median of 18 days (range, 9–34). The remaining eight pigs (*n* = 8; Acute_inf group) were euthanized after exhibiting mucohemorrhagic diarrhea for two consecutive days, which occurred at a median of 20 days (range, 7–34). Non-infected pigs (*n* = 16; control group) were euthanized and necropsied in parallel with their infected counterparts. During necropsy, the apex region of the colon was identified, and its contents were collected by incision, immediately snap-frozen in liquid nitrogen, and stored at −80℃ until further processing. However, two pigs initially allocated to the Early_inf group were excluded from the study, as the colon content obtained at necropsy was insufficient to carry out the downstream analyses.

### Untargeted metabolomics and data processing

Untargeted metabolomics analysis was carried out by following the methodology used by López-Bascón et al. [17]. A total of 150 mg of colon content sample was weighed in glass tubes for extraction of metabolites with 4 mL of methanol. The suspension was agitated for 5 min, and then, the phases were separated by centrifugation at 12,500* × g* for 10 min at 4 ℃. The supernatant was filtered through a 0.2-μm filter to remove suspended particles.

Liquid chromatography–tandem mass spectrometry (LC–MS/MS) and gas chromatography–mass spectrometry (GC–MS) analyses were carried out by application of the methods described by López-Bascón et al. [17]. For LC–MS/MS analysis, 100 μL of the extract was evaporated to dryness and reconstituted with 100 μL of acetonitrile by shaking in a vortex for 30 s. The analysis was carried out with an Agilent 1200 Series LC system coupled to an Agilent 6540 UHD QTOF mass spectrometer (Agilent Technologies, USA). For GC–MS analysis, 100 μL of the extract was evaporated to dryness and directly reconstituted in the specified volume for methoxymation and further silylation. An Agilent 7890A Series GC system coupled to an Agilent 7200 UHD QTOF mass spectrometer was used for this purpose.

Quality controls (QCs), prepared as a pool of all samples, were analyzed daily (three QCs per day) to detect sources of potential variability associated with the sequence of analyses. Relative abundances were estimated in both LC−MS/MS and GC–MS analyses by applying the Mass Spectrometry Total Useful Signals (MSTUS) strategy [18] to minimize the analytical variability.

### Bioinformatic analysis

Potential differences in metabolite profiles were analyzed by comparing samples from infected and non-infected pigs, as well as by comparing samples across the three experimental groups according to their stage of infection (control, Early_inf, and Acute_inf).

On the basis of the relative abundance of metabolites, relationships among samples were analyzed and visualized using the vegan version 2.6–4 [19] and ggplot2 version 3.5.1 [20] packages in R [21]. Samples were ordinated using Principal Coordinate Analysis (PCoA) on the basis of Bray–Curtis distances calculated with the vegdist function. Dissimilarities between groups were tested with a permutational multivariate analysis of variance (PERMANOVA), using the adonis2 and pairwiseAdonis functions (pairwiseAdonis version 0.4.1 R package [22]). Within-group dispersion was evaluated using the betadisper function by comparing the distances of samples to their respective group centroids with the Wilcoxon test. The influence of metabolites and experimental variables on the ordination was evaluated by fitting linear models to the ordination results using the envfit function. *p*-values were adjusted using the Benjamini–Hochberg (BH) procedure, and adjusted *p*-values ≤ 0.05 were considered statistically significant.

Data were log-transformed (log_10_) and normalized by centering the mean and dividing by the standard deviation of each variable. Hierarchical clustering was based on Euclidean distance measure and ward clustering algorithm. Univariate analysis was performed by an independent *t*-test based on the limma R package. Adjusted *p*-values ≤ 0.05 were considered statistically significant. The capability to discriminate between sample groups on the basis of the metabolite composition was tested ten times using the random forest algorithm. The abovementioned methodology was implemented in the online tool MetaboAnalyst 6.0 [23].

### Multiomics data integration

Data Integration Analysis for Biomarker Discovery Using Latent Variable Approaches for Omics Studies (DIABLO) based on the sparse Partial Least Squares Discriminant Analysis (sPLS-DA) framework from the mixOmics version 6.28.0 R package [24, 25] was used to integrate the metabolomic and metagenomics data and to discriminate between the experimental groups (control, Early_inf, and Acute_inf). The metagenomic data were already published in a previous study performed with the same animals [7]. Bacterial species present in fewer than 20% of the samples were filtered out. The metagenomic data were transformed using the centered log-ratio (CLR) approach (compositions version 2.0–9 R package [26]), and the metabolomic data were median-normalized [23]. These processed datasets were then used in the supervised integration analysis, allowing the selection of 40 bacterial species and 40 metabolites.

Model performance was evaluated using fivefold cross-validation repeated ten times with the perf function, and prediction accuracy was assessed on the left-out samples for each block (metabolomic dataset, taxonomy dataset) using receiver operating characteristic (ROC) curves and area under the curve (AUC) plots generated with the auroc function [24].

Finally, a Circos plot was constructed on the basis of a similarity matrix to explore inter-block relationships and interactions, applying a correlation threshold of 0.7 to identify strong associations [24].

## Results

### Metabolomic profiles of colonic contents vary with the severity of *Brachyspira hyodysenteriae* infection

**Figure 1:**
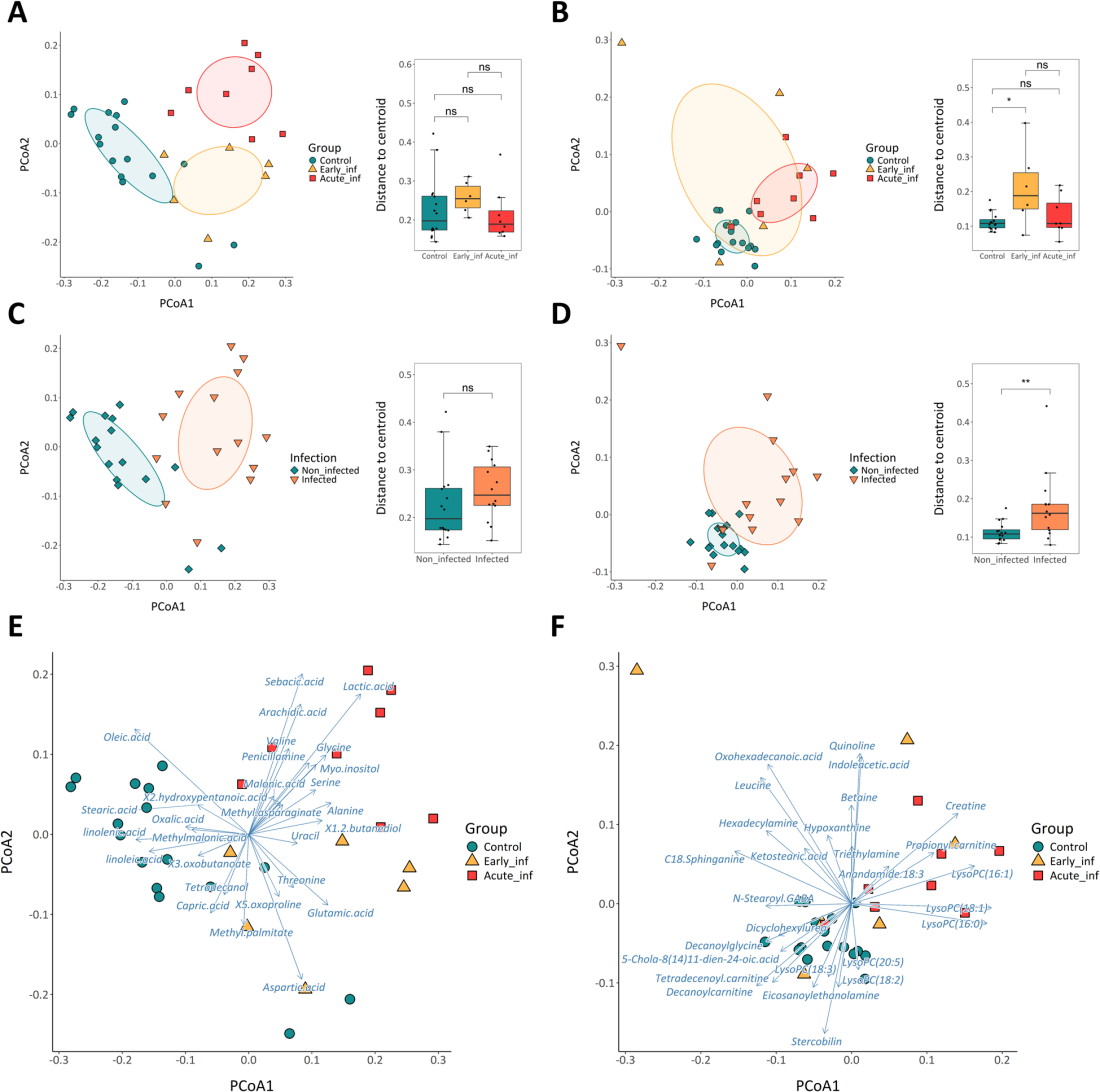
**Beta-diversity analysis of the detected metabolites by A,** **C,** **E GC–MS and B,** **D,** **F LC–MS/MS in colonic content samples based on Bray–Curtis distances and distributed in a principal coordinate analysis (PCoA)**. Data are represented by **A**, **B** variable groups and **C**, **D** infection. The ellipses represent covariance of each variable under study. Box plots display the distance to the centroid of each variable under study. **E**, **F** The main metabolites influencing sample ordination returned by the envfit model with significant BH-adjusted *p*-value are represented by blue arrows. Arrow lengths show the strength of each metabolite influencing the ordination of samples. Not significant (ns) *p* > 0.05, ^*^p ≤ 0.05, ^**^p ≤ 0.01, ^***^p ≤ 0.001 (Wilcoxon test or ANOVA and Tukey tests, with Holm adjustment).

A total of 47 and 76 metabolites were identified by untargeted metabolomic analysis performed with GC–MS (Additional File 1) and LC–MS/MS (Additional File 2), respectively. Sample ordination based on Bray–Curtis distances and plotted into a PCoA revealed differences in the metabolomic profiles by group. Samples from the Acute_inf group were clearly separated from the control group (*p* < 0.01) (Table 1 and Figure 1A, B). In contrast, samples from the Early_inf group were dispersed between these two groups. Although significant differences were also observed between the Early_inf group and both the control (*p* < 0.05) and Acute_inf (*p* < 0.01) groups, indicating an intermediate metabolic profile, the absence of differences in the metabolite profiles identified by the LC–MS/MS method between the Early_inf and Acute_inf groups suggests a closer metabolic similarity between these two groups (Table 1). This similarity contributed to the overall significant differences observed between infected and non-infected pigs (*p* < 0.001) (Figure 1C, D and Table 1). Sample distribution by infection group seems to have a higher association with metabolites in GC–MS than LC–MS/MS analyses. This observation may also reflect a lower analytical sensitivity of LC–MS/MS resulting from the more limited development of databases for this tecnique compared with GC–MS. In addition, samples from the Early_inf group showed greater dispersion, contributing to the significant differences in dispersion observed between non-infected and infected pigs (*p* < 0.01) as determined by the metabolite profiles identified using the LC–MS/MS method (Figure 1B, D). The metabolites influencing the sample distribution in GC–MS and LC–MS/MS analyses are shown in Figure 1E, F.

**Table 1:** **Influence of metabolites on ordination of samples and results of the permutational multivariate analysis of variance based on Bray–Curtis distances**

GC–MS				
Factor	envfit		PERMANOVA	
	*R*^2^	*p*-Value	*R*^2^	*p*-Value
Infection	0.5062	0.001^***^	0.2385	0.001^***^
Group	0.6452	0.001^***^	0.0661	0.022^*^
Control versus Early_inf	–	–	0.1917	0.006^**^
Control versus Acute_inf	–	–	0.2992	0.003^**^
Early_inf versus Acute_inf	–	–	0.1680	0.012^*^

LC–MS/MS				
Factor	envfit		PERMANOVA	
	*R*^2^	*p*-Value	*R*^2^	*p*-Value
Infection	0.3443	0.001^***^	0.1395	0.001^***^
Group	0.4382	0.001^***^	0.0828	0.007^**^
Control versus Early_inf	–	–	0.1197	0.024^*^
Control versus Acute_inf	–	–	0.2682	0.003^**^
Early_inf versus Acute_inf	–	–	0.1343	0.174

^*^ *p* ≤ 0.05, ^**^p ≤ 0.01, ^***^p ≤ 0.001, ^****^p ≤ 0.0001

### Aminoacids, carnitines, and saturated lyso-phosphatidylcholines define the SD metabolome

**Figure 2:**
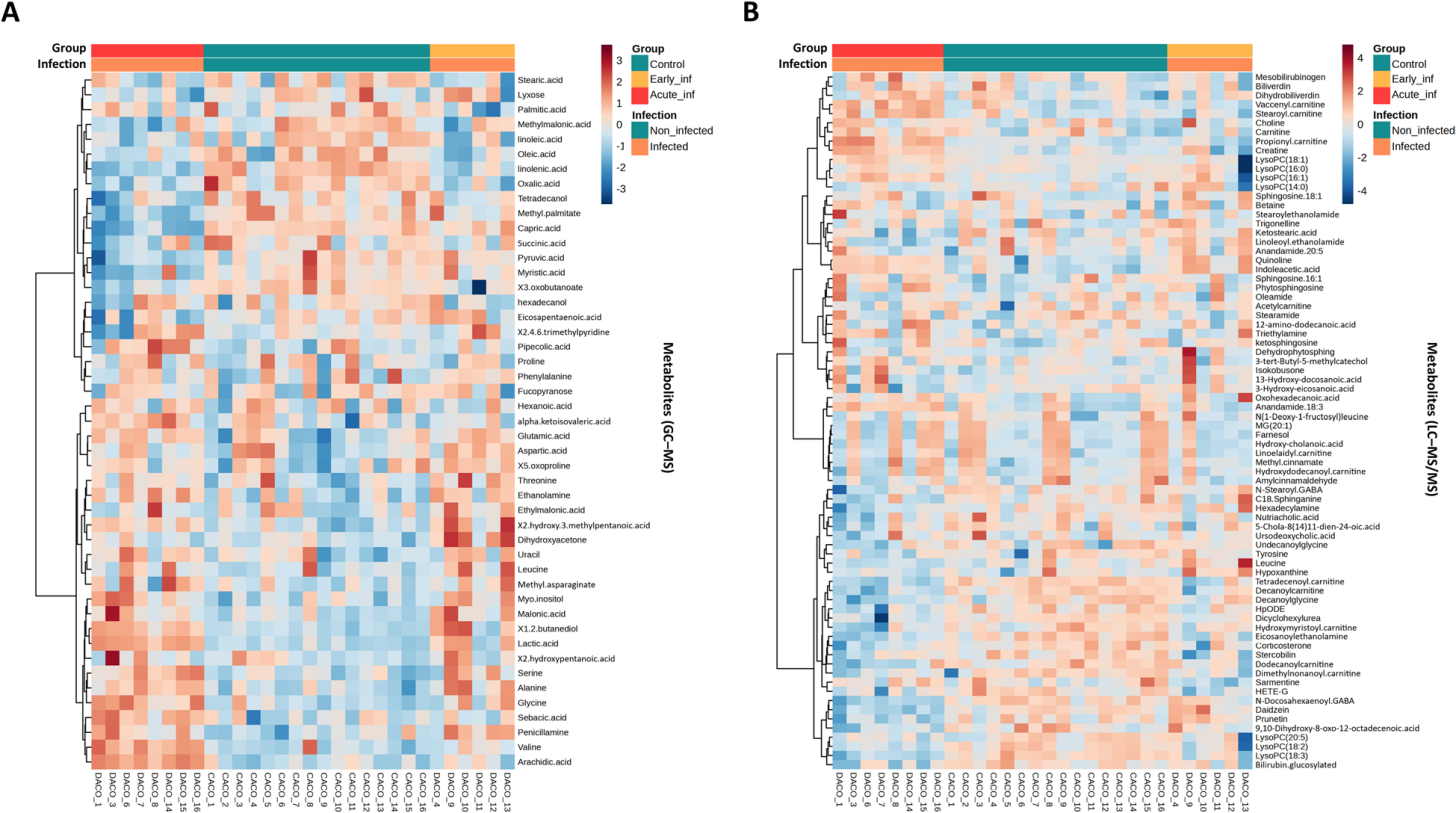
**Heat map of the normalized abundance of metabolome data from A GC–MS and B LC–MS/MS in colonic content samples.** The hierarchical clustering reveals sample clustering by metabolites and, at the top, according to the group and infection variables.

The hierarchical clustering of samples based on Euclidean distances also grouped the samples by the infection groups, evidencing which metabolites were altered in the colon content from Acute_inf pigs compared with non-infected controls. However, profiles from Early_inf samples share similar features with both Acute_inf and control samples (Figure 2). The clustering was defined by a higher concentration of amino acids (i.e., valine, glycine, alanine, and serine), arachidic acid, 1,2-butanediol, and lactic acid in Acute_inf samples and capric acid and methyl palmitate in controls for the GC–MS dataset (Figure 2A). In Acute_inf samples, the LC–MS/MS analysis reported a higher proportion of carnitine and its acyl derivatives (vaccenyl carnitine, stearoyl carnitine, and propionyl carnitine) as well as lyso-phosphatidylcholines (LysoPC) 18:1, LysoPC 16:0, LysoPC 16:1, and LysoPC 14:0. Otherwise, control samples exhibited higher concentrations of LysoPC 18:3, LysoPC 18:2, and LysoPC 20:5, *N*-docosahexaenoyl, decanoyl carnitine, tetradecenoyl carnitine, and, to a lesser extent, dimethylnonanoyl carnitine, dicyclohexylurea, and decanoylglycine (Figure 2B). Several of these metabolites significantly contributed to the ordination of samples by group in the PCoA plot (Figure 1E, F).

### *Brachyspira hyodysenteriae* infection increases amino and organic acids while decreasing fatty acid concentrations in colon contents

**Figure 3:**
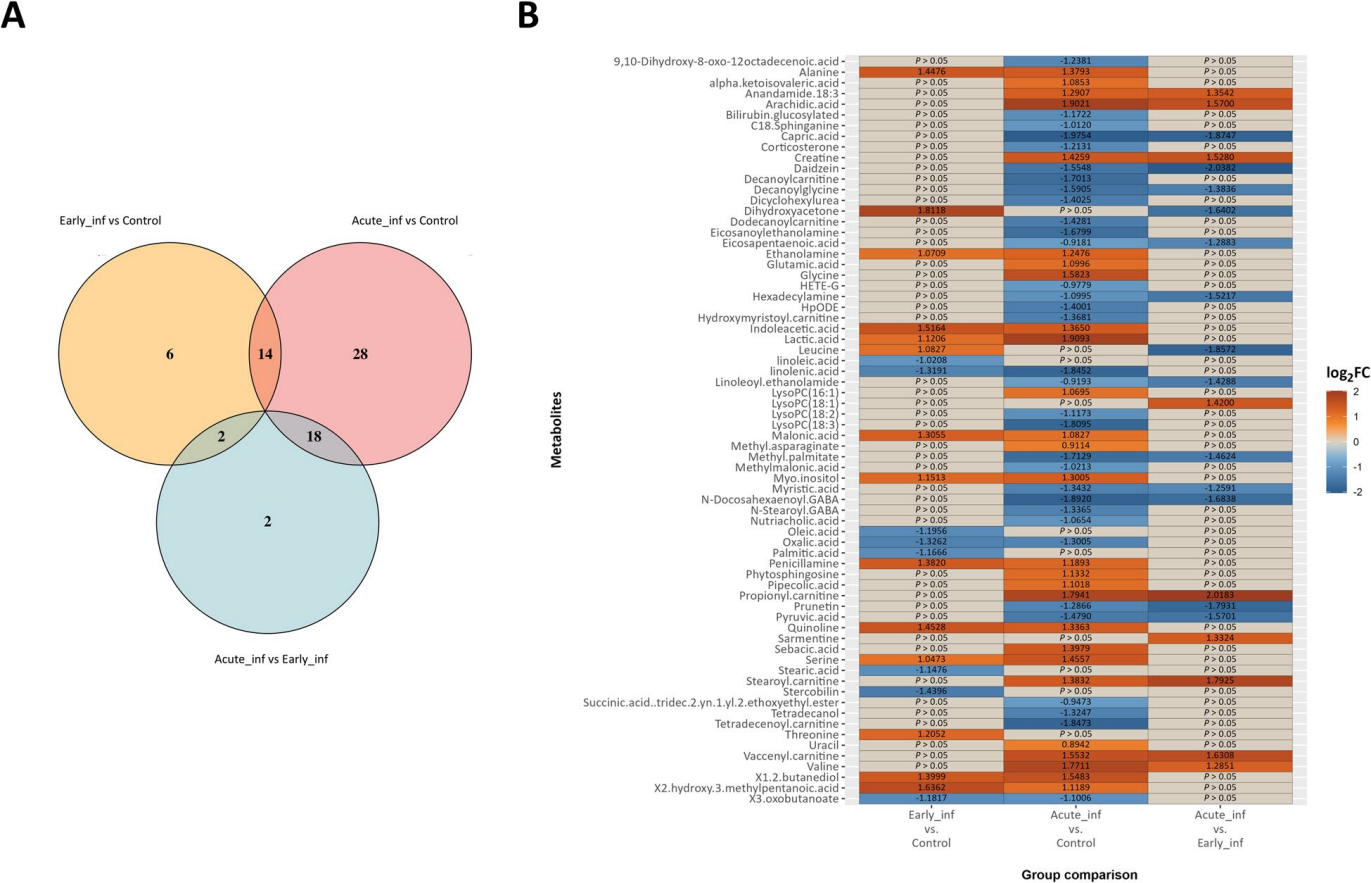
**A** Venn diagram summarizing the unique and shared metabolites (*p* ≤ 0.05) between factorial analysis of differences based on the group variable. **B** Heat map visualization of the log_2_ fold-change values of differentially abundant metabolites between categories in the group variable. Significant threshold is established at *p* ≤ 0.05.

An analysis of differentially abundant metabolites was performed by a linear model. This analysis revealed that 60 compounds varied significantly (*p* ≤ 0.05) in the Acute_inf group compared with the control group for both datasets. Furthermore, 14 of them also exhibited significant differences between control and Early_inf (Figure 3A and Additional Files 3 and 4). Among these compounds, there was an increase of amino acids (alanine and serine), several organic acids (indoleacetic acid, lactic acid, malonic acid, 2-hydroxy-3-methylpentanoic acid), amines (ethanolamine, penicillamine), and other compounds such as myo-inositol, quinoline, and 1,2-butanediol. However, fatty acids such as linolenic acid and oxalic acid were found to be significantly decreased along with 3-oxobutanoate (Figure 3B and Additional Files 3 and 4).

However, 18 common metabolites were statistically significant when comparing the Acute_inf group with the Early_inf and control groups (Figure 3A). Among the altered metabolites, we observed an increase in fatty acids and derivatives such as anandamide 18:3 and arachidic acid, creatine, acylcarnitines (propionyl carnitine, stearoyl carnitine, vaccenyl carnitine), and the amino acid valine. In contrast, a decrease was observed in fatty acids such as capric acid, decanoylglycine, eicosapentaenoic acid, methyl palmitate, myristic acid, and pyruvic acid, as well as in compounds including isoflavones (daidzein, prunetin), amines (hexadecylamine, linoleoyl ethanolamide), and gamma-aminobutyric acid (GABA) derivates (*N*-docosahexaenoyl GABA) (Figure 3B and Additional Files 3 and 4).

### The intestinal metabolome predicts the infection status in SD-infected pigs

**Figure 4:**
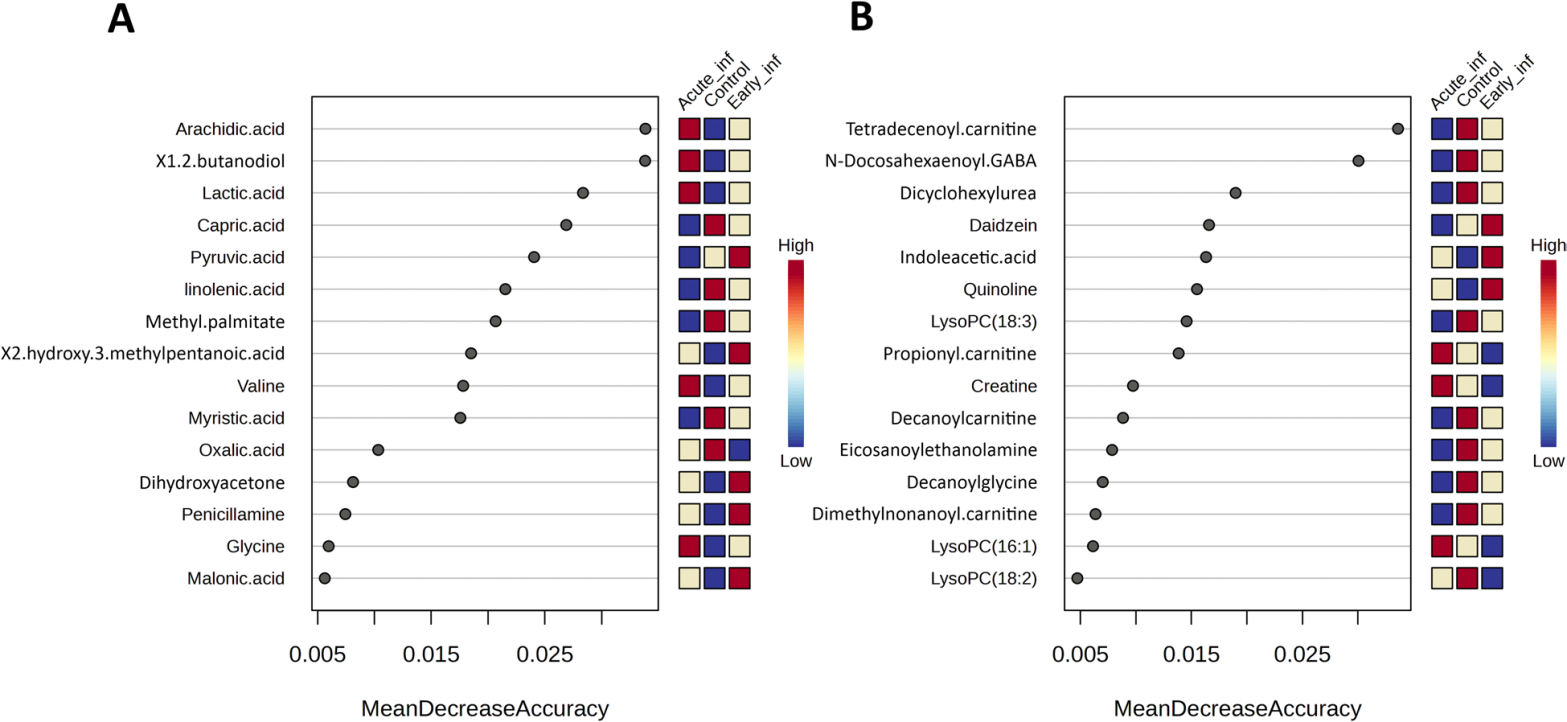
**Random forest variable importance plot summarizing the infection group prediction by different metabolites obtained using A GC–MS and B LC–MS/MS analysis.** Group prediction for each metabolite is depicted from red to blue, while the higher mean decrease accuracy values provide a reference of the relevance of that metabolite in predicting the group.

A random forest algorithm was used to model and predict the classification of samples into the three groups included in the study. The model was able to correctly classify the samples into their respective group with a mean out-of-bag error of 0.14 and 0.26 for GC–MS and LC–MS datasets, respectively (Additional File 5). While matchings for the control and the Acute_inf groups were accurate, Early_inf samples were misplaced in control (GC–MS and LC–MS/MS dataset) or Acute_inf (LC–MS/MS dataset) groups (Additional File 5). Sample discrimination was mainly associated with arachidic acid, 1,2-butanediol, lactic acid, capric acid, pyruvic acid, linolenic acid, and methyl palmitate compounds for the GC–MS dataset (Figure 4A) and tetradecenoyl carnitine and *N*-docosahexaenoyl GABA for the LC–MS/MS dataset (Figure 4B).

### Multiomics integration reveals metabolite–microbiota interactions, underscoring profiles associated with SD

**Figure 5:**
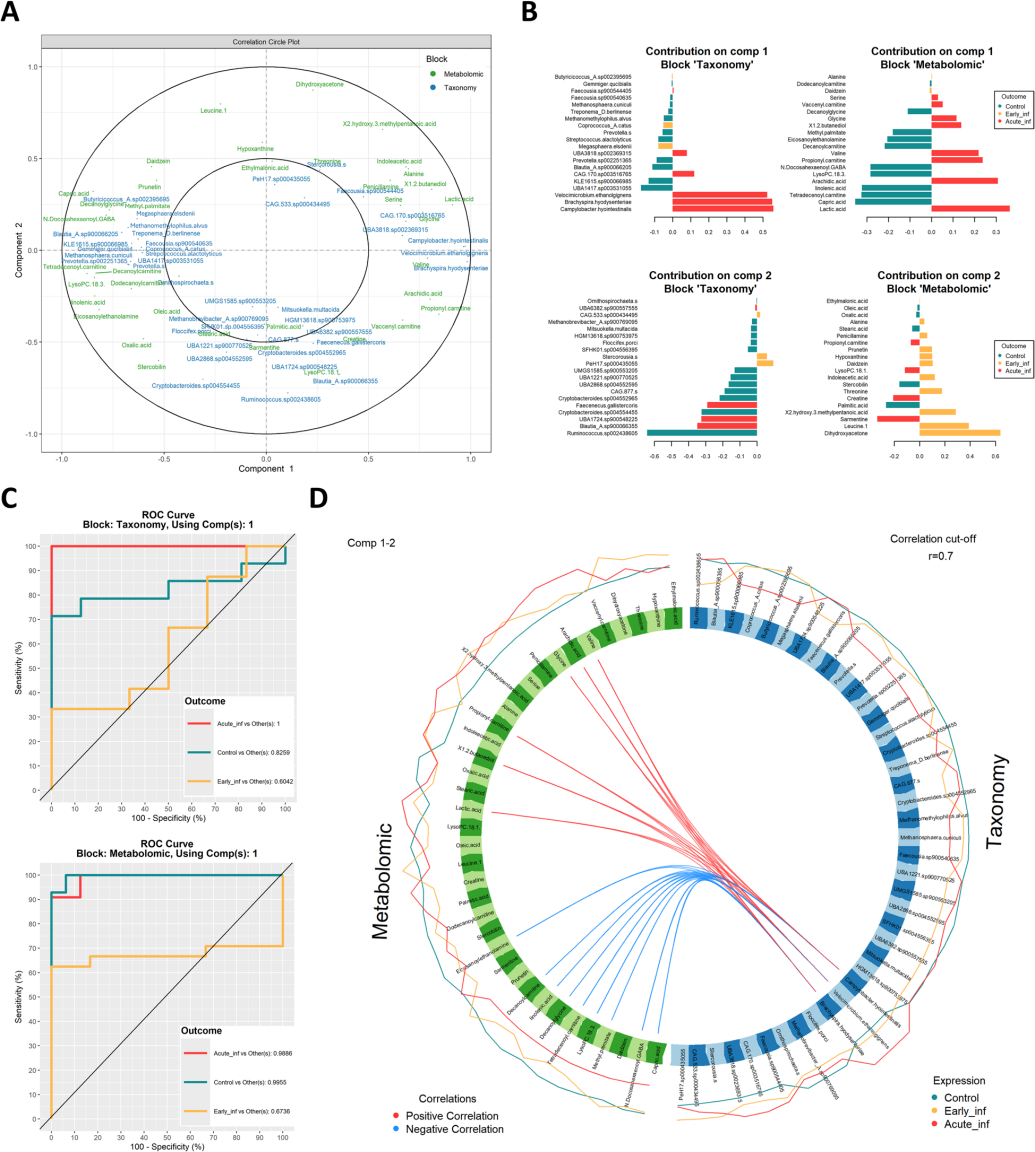
**Multiomics integration of metabolomic and metagenomic data from colonic contents analyzed with block sPLS-DA.** **A** Correlation circle plot combining metabolic (green) and shotgun metagenomic (blue) datasets. **B** Loading values for mean contributors for each group (control, Early_inf, and Acute_inf) for taxonomy (left) and metabolites (right). **C** ROC curve for performance assessment of the final block sPLS-DA model for taxonomy (upper) and metabolites (lower) using component 1 in the three categories for the group variable. **D** Circos plot displaying correlations (cut-off = 0.7) between metabolomic (green) and metagenomic (blue) datasets. Red and blue lines denote positive and negative correlations, respectively. Outer lines represent the abundances of each taxon/metabolite for each group.

The integrative analysis of the metabolomic and metagenomic datasets revealed a strong overall correlation (*R* = 0.96), once again showing an intermediate dispersion of the Early_inf group between Acute_inf and control. The analysis identified a signature comprising 40 bacterial species and 37 metabolites that contributed most to group discrimination (Figure 5A). Lactic acid and arachidic acid were the main discriminant metabolites for the Acute_inf group, whereas capric acid and tetradecenoyl carnitine were features characteristic of the control group. Finally, dihydroxyacetone and leucine were most distinctive metabolites associated with the Early_inf group (Figure 5B). Taxonomically, *Brachyspira hyodysenteriae* and *Campylobacter hyointestinalis* were predominant discriminant species in the Acute_inf group, *Ruminococcus* sp. 002438605 and *Crystobacteroides* sp. 004554455 in the control group, and *Megasphaera elsdenii* in the Early_inf group (Figure 5B).

The predictive performance of the selected variables was evaluated using the area under the receiver operating characteristic curve (AUROC). Both the metabolomics and taxonomy blocks demonstrated high predictive power for disease status classification (Figure 5C). Considering the 20 variables selected in component 1, the metabolite block achieved AUROC values of 0.989, 0.674, and 0.996 for the Acute_inf, Early_inf, and control groups, respectively, while the taxonomic block yielded AUROC values of 1.000, 0.604, and 0.826 for the same groups. Metabolites showed a comparable yet slightly better performance than taxonomic features in predicting SD during its early stages.

The strongest correlations between the selected colonic metabolites and microbial species are illustrated in a Circos plot (Figure 5D). Bacterial species enriched in the Acute_inf group, including *Brachyspira hyodysenteriae*, *Velocimicrobium ethanolgignens*, and *Campylobacter hyointestinalis* exhibited strong positive correlations (*R* > 0.7) with metabolites previously associated with this group, such as valine, arachidic acid, glycine, propionyl carnitine, 1,2-butanodiol, and lactic acid. Conversely, these species showed negative correlations (*R* < 0.7) with eicosanoylethanolamine, decanoylcarnitine, linolenic acid, decanoylglycine, tetradecenoyl carnitine, LysoPC 18:3, methyl palmitate, *N*-docosahexaenoyl GABA, and capric acid.

## Discussion

Necrosis of the colonic epithelial barrier, inflammation, exacerbated mucin production, or displacement of the commensal microbiota are characteristic features of SD [5, 27, 28]. In recent years, knowledge on the host response to the infection and the changes in the gut microbiota are being disclosed [7, 8, 29, 30], but little is known about the metabolic context of SD in the gut. Here, for the first time, using an in vivo model, we describe the impact of these changes in the colon metabolome and provide a snapshot of the intestinal environment during early and acute SD infection.

Untargeted metabolomic analysis is a powerful tool to characterize with precision the metabolites present in feces, as previously reported [17]. The combination of untargeted metabolomics with the design of our in vivo challenge revealed that the colonic metabolome suffered progressive changes in the *B. hyodysenteriae* infection. Compared with the non-infected controls, the Early_inf group exhibited an intermediate metabolome profile that progressed to an altered profile during the acute infection stage. The metabolic features of acute infection were so characteristic that they even enabled the prediction of the Acute_inf group pigs using random forest analysis based on their metabolome profiles.

The metabolome of the infected groups was characterized by increased levels of amino acids, particularly alanine and serine. Amino acids accumulation in colonic contents may suggest intestinal malabsorption [31, 32] or leaky gut, as previously mentioned in relation to valine [33, 34]. An increase in serine during acute infection may be associated with the proliferation of colonocytes and immune cells in response to mucosal damage [35], and to the overstimulation of mucin production elicited by the infection [36]. The potential deficiencies in amino acid associated with the infection could negatively impact the energy metabolism [35, 37, 38] and impair the immune response, limiting lymphocyte proliferation [35, 38].

The untargeted metabolomics detected a substantial number of fatty acids or their derivatives altered by the infection. Indeed, a medium-chain fatty acid (MCFA), capric acid, was the metabolite that better predicted the control group in the random forest analysis performed. This fatty acid is linked to gut health through several mechanisms [39, 40], and its alteration reflects the disruption of physiological processes in the colon.

Proinflammatory metabolites reduce the uptake of carnitine, a molecule with recognized anti-inflammatory properties as an immune regulator [41], thereby impairing epithelial cell repair and regeneration processes [42]. The decrease of carnitine absorption can explain the increase of carnitine concentration in the metabolomic dataset for individuals with acute infection. The combination of fatty acids and carnitine generates a variety of molecules known as acylcarnitines, whose chain lengths influence their chemical structure and metabolic function [43]. These molecules participate in processes such as energy metabolism, mitochondrial homeostasis, or inflammation, among others [44]. Our study revealed increased concentration of carnitine and long-chain acylcarnitine (LCAC) (i.e., propionyl carnitine, stearoyl carnitine, and vaccenyl carnitine) and decreased concentration of decanoyl carnitine and tetradecanoyl carnitine in the Acute_inf group. Previous studies have revealed that high concentration of LCACs can damage mitochondrial function [45, 46] and result in the increase of reactive oxygen species (ROS) production [47], stress the endoplasmic reticulum, and accelerate cell damage [48].

Ethanolamine and acylethanolamides (AEs), molecules derived from long-chain fatty acids, were increased in infected pigs. Ethanolamine is a component of phospholipids in cell membranes and is abundant in the intestinal tract owing to dietary intake, exfoliation of enterocytes, and the turnover of bacterial cells [49, 50]. It is an important signaling molecule whose availability increases under intestinal inflammation [51]. Interestingly, this metabolite serves as an energy source for various intestinal pathogens, such as *Salmonella* Typhimurium, enterohemorrhagic *Escherichia coli* (EHEC), *Enterococcus faecalis*, and *Clostridioides difficile*, thus gaining a competitive advantage over the resident microbiota, which does not readily metabolize this compound [49, 51, 52]. In the Acute_inf group, we observed increased abundance of anandamide (arachidonoylethanolamine), one of the most extensively studied AEs. Its concentration is increased in inflammatory bowel disease (IBD), where it is thought to represent an adaptive protective response to inflammation, modulating the immune system by reducing proinflammatory cytokine production, epithelial damage, and intestinal motility [53, 54]. Several studies have shown that luminal AEs concentrations in inflamed gut correlate with shifts in the relative abundance of specific bacterial taxa, potentially contributing to disease pathogenesis [55–57]. Anandamide derives from arachidonic acid, whose hydrogenation results in arachidic acid [58, 59]. The molecule, detected at higher concentrations in acutely infected animals, is associated with tissue injury or irritation, by the release of omega-6-related metabolites from cell membranes, acting as proinflammatory mediators and precursors of signaling molecules such as eicosanoids [60]. Furthermore, omega-3 fatty acids and derivatives such as linolenic acid, eicopentaenoic acid, and *N*-docosahexaenoyl GABA with anti-inflammatory activity [61, 62] decrease as the disease develops.

LysoPC metabolites, particularly the monounsaturated LysoPC 16:1 and the polyunsaturated LysoPC 18:2 and LysoPC 18:3 were depicted as drivers of the group arrangement in the PCoA. Interestingly, the polyunsaturated species (LysoPC 18:2, LysoPC 18:3, and LysoPC 20:5), attributed to anti-inflammatory effects [12], featured the metabolomic profile of control pigs. Conversely, the saturated (LysoPC 16:0 and LysoPC 18:0) and monounsaturated (LysoPC 18:1) metabolites, highly abundant in Acute_inf samples, are related to proinflammatory response in the host [63–65]. These metabolites are known to be present in blood [66], and their higher concentration could be linked to the hemorrhagic diarrhea experienced by these animals.

The Acute_inf group exhibited higher concentration of colonic creatine. This contrasts with previous studies in which creatine, together with glutamic acid, showed lower levels in colon explants exposed to *B. hyodysenteriae* compared with controls, measured only 8 h after exposure [4]. Differences in the matrix used (explants versus in vivo intestine) and in samples collection timing due to infection progression could explain the discrepancies observed between studies. Interestingly, we previously detected high levels of creatine kinase in serum samples from the Acute_inf group [6]. The increase in creatine kinase in plasma is related to mitochondrial dysfunction and inflammatory bowel diseases [67–69]. Creatine has a relevant role in enhancing ATP in high-demand circumstances [70] through the transfer of a phosphoryl group to re-form ATP involving creatine kinase [71]. Thus, it could be hypothesized that the creatine metabolism plays a role in the supply of energy in Acute_inf individuals due to its deficiency caused by intestinal epithelium damage. Other metabolites that were increased during the acute infection were indol derivates, such as indoleacetic acid, already proposed as stress biomarker [72], 1,2-butanediol, which may evidence a shift in the fermentation process in the large intestine, and osmolyte myo-inositol, potentially associated with hyperosmotic stress, enhancing the diarrhea process. Besides, we reported an elevated concentration of lactic acid in the infected groups, in agreement with the results obtained in previous studies with patients with IBD [73–75]. The observed dysbiosis in the colon of these animals [7] may be associated with lactate accumulation by impairing the conversion of lactate into butyrate, leading to increased levels of lactic acid in the colonic lumen [75]. Lactate has also been associated with risk of diarrhea and mucosal inflammation [76], as glycolysis (transformation of glucose into pyruvate and energy) is promoted by chronic inflammation [77].

In a previous study using the colonic content from the same animals, we observed that SD significantly altered the gut microbiota [7]. Here we integrated metabolomic and metagenomic databases to evaluate potential interactions. The integrative analyses allowed for the selection of 40 bacterial species and 37 metabolites that contributed to infection group discrimination. The global results suggested a possible bacteria-driven metabolomic profile, mostly associated with not only the increased relative abundance of *B. hyodysenteriae* but also *C. hyointestinalis* and *V. ethanolgignens* in acutely infected pigs. The metabolic shift from healthy controls to SD pigs includes a decrease in metabolites with anti-inflammatory functions, such as tetradecanoyl and capric acid, and an increase in metabolites associated with tissue damage and intestinal inflammation, such as propionyl carnitine and lactic acid.

Interestingly, our integrative model identified leucine and dihydroxyacetone as potential biomarkers for predicting early stage SD. Disruption in the integrity of the mucosa could result in the leakage of amino acids (i.e., leucine) [78] into the lumen. In addition, dihydroxyacetone acts as a glycolysis intermediate associated with the enrichment of proinflammatory or opportunistic bacterial species [79]. An increase in dihydroxyacetone is linked to increased oxidative stress and could contribute to disease progression [80].

## Conclusions

Our results demonstrate that *B. hyodysenteriae* infection induces progressive alterations in the colonic metabolome of pigs, characterized by a reduction in metabolites with anti-inflammatory properties and increased concentrations of compounds linked to tissue damage. These metabolic changes likely contribute to the development of the characteristic lesions and clinical outcome of SD. Moreover, the study identifies specific metabolites with potential as predictive biomarkers for early disease detection. Overall, these findings provide a robust foundation for future research aimed at elucidating the mechanistic links between bacterial species, metabolic alterations, and disease progression, ultimately enhancing strategies for the prevention and treatment of SD.

## Supplementary Information

**Additional file 1. Relative abundance of metabolites detected by GC–MS method.**

**Additional file 2. Relative abundance of metabolites detected by LC–MS/MS method.**

**Additional file 3. Linear model results for differential abundance metabolites detected by GC–MS method.**

**Additional file 4. Linear model results for differential abundance metabolites detected by LC–MS/MS method.**

**Additional file 5. Classification of the samples by “Group” computed 10 times by random forest learning method.**

## Data Availability

the datasets generated and analyzed during the current study are available from the corresponding author upon reasonable request.

## Abbreviations

AEs: acylethanolamides
ANOVA: analysis of variance
ATCC: American Type Culture Collection
ATP: adenosine triphosphate
AUC: area under the curve
AUROC: area under the receiver operating characteristic curve
BH: Benjamini–Hochberg
CLR: centered log-ratio
DIABLO: data integration analysis for biomarker discovery using latent variable approaches for omics studies
EHEC: enterohemorrhagic *Escherichia coli*
GABA: gamma-aminobutyric acid
GC–MS: gas chromatography–mass spectrometry
IBD: inflammatory bowel disease
LCAC: long-chain acylcarnitine
LC–MS/MS: liquid chromatography–tandem mass spectrometry
LysoPC: lyso-phosphatidylcholines
MCFA: medium-chain fatty acid
MSTUS: mass spectrometry total useful signals
PCoA: principal coordinate analysis
PERMANOVA: permutational multivariate analysis of variance
QCs: quality controls
ROC: receiver operating characteristic
SD: swine dysentery
sPLS-DA: sparse Partial Least Squares Discriminant Analysis
