# Supplementary material for: Metabolomic signatures of colonic infection by Brachyspira hyodysenteriae
Source: Vet Res. 2026 May 19;57:78. doi: 10.1186/s13567-026-01727-9 (PMC13188474; doi:10.1186/s13567-026-01727-9)
Supplement: Supplementary file 5 — Additional file 5. Classification of the samples by “Group” computed 10 times by random forest learning method. [file 13567_2026_1727_MOESM5_ESM.docx]

| **GC–MS** | | | | | | | | | | | | | |
| --- | --- | --- | --- | --- | --- | --- | --- | --- | --- | --- | --- | --- | --- |
| Sample | Classified as | Test1 | Test2 | Test3 | Test4 | Test5 | Test6 | Test7 | Test8 | Test9 | Test10 | Mean | SD |
| Control | Control | 15.00 | 15.00 | 15.00 | 15.00 | 15.00 | 15.00 | 15.00 | 15.00 | 15.00 | 15.00 | 15.00 | 0.00 |
| Control | Early_inf | 1.00 | 1.00 | 1.00 | 1.00 | 1.00 | 1.00 | 1.00 | 1.00 | 1.00 | 1.00 | 1.00 | 0.00 |
| Control | Acute_inf | 0.00 | 0.00 | 0.00 | 0.00 | 0.00 | 0.00 | 0.00 | 0.00 | 0.00 | 0.00 | 0.00 | 0.00 |
| Early_inf | Early_inf | 3.00 | 3.00 | 3.00 | 3.00 | 3.00 | 3.00 | 3.00 | 3.00 | 3.00 | 3.00 | 3.00 | 0.00 |
| Early_inf | Control | 3.00 | 3.00 | 3.00 | 3.00 | 3.00 | 3.00 | 3.00 | 3.00 | 3.00 | 3.00 | 3.00 | 0.00 |
| Early_inf | Acute_inf | 0.00 | 0.00 | 0.00 | 0.00 | 0.00 | 0.00 | 0.00 | 0.00 | 0.00 | 0.00 | 0.00 | 0.00 |
| Acute_inf | Acute_inf | 8.00 | 7.00 | 8.00 | 8.00 | 7.00 | 8.00 | 8.00 | 7.00 | 8.00 | 8.00 | 7.70 | 0.48 |
| Acute_inf | Control | 0.00 | 0.00 | 0.00 | 0.00 | 0.00 | 0.00 | 0.00 | 0.00 | 0.00 | 0.00 | 0.00 | 0.00 |
| Acute_inf | Early_inf | 0.00 | 1.00 | 0.00 | 0.00 | 1.00 | 0.00 | 0.00 | 1.00 | 0.00 | 0.00 | 0.30 | 0.48 |
| Control | class.error | 0.06 | 0.06 | 0.06 | 0.06 | 0.06 | 0.06 | 0.06 | 0.06 | 0.06 | 0.06 | 0.06 | 0.00 |
| Early_inf | class.error | 0.50 | 0.50 | 0.50 | 0.50 | 0.50 | 0.50 | 0.50 | 0.50 | 0.50 | 0.50 | 0.50 | 0.00 |
| Acute_inf | class.error | 0.00 | 0.13 | 0.00 | 0.00 | 0.13 | 0.00 | 0.00 | 0.13 | 0.00 | 0.00 | 0.04 | 0.06 |
| Out-of-bag | OOB | 0.13 | 0.17 | 0.13 | 0.13 | 0.17 | 0.13 | 0.13 | 0.17 | 0.13 | 0.13 | 0.14 | 0.02 |
| **LC–MS/MS** | | | | | | | | | | | | | |
| Sample | Classified as | Test1 | Test2 | Test3 | Test4 | Test5 | Test6 | Test7 | Test8 | Test9 | Test10 | Mean | SD |
| Control | Control | 15.00 | 15.00 | 15.00 | 15.00 | 15.00 | 14.00 | 15.00 | 15.00 | 15.00 | 15.00 | 14.90 | 0.32 |
| Control | Early_inf | 0.00 | 0.00 | 0.00 | 0.00 | 0.00 | 1.00 | 0.00 | 0.00 | 0.00 | 0.00 | 0.10 | 0.32 |
| Control | Acute_inf | 1.00 | 1.00 | 1.00 | 1.00 | 1.00 | 1.00 | 1.00 | 1.00 | 1.00 | 1.00 | 1.00 | 0.00 |
| Early_inf | Early_inf | 0.00 | 0.00 | 0.00 | 0.00 | 0.00 | 1.00 | 1.00 | 0.00 | 1.00 | 0.00 | 0.30 | 0.48 |
| Early_inf | Control | 4.00 | 4.00 | 4.00 | 4.00 | 4.00 | 4.00 | 4.00 | 4.00 | 4.00 | 4.00 | 4.00 | 0.00 |
| Early_inf | Acute_inf | 2.00 | 2.00 | 2.00 | 2.00 | 2.00 | 1.00 | 1.00 | 2.00 | 1.00 | 2.00 | 1.70 | 0.48 |
| Acute_inf | Acute_inf | 7.00 | 7.00 | 7.00 | 7.00 | 7.00 | 8.00 | 7.00 | 7.00 | 7.00 | 7.00 | 7.10 | 0.32 |
| Acute_inf | Control | 1.00 | 1.00 | 1.00 | 1.00 | 1.00 | 0.00 | 1.00 | 1.00 | 1.00 | 1.00 | 0.90 | 0.32 |
| Acute_inf | Early_inf | 0.00 | 0.00 | 0.00 | 0.00 | 0.00 | 0.00 | 0.00 | 0.00 | 0.00 | 0.00 | 0.00 | 0.00 |
| Control | class.error | 0.06 | 0.06 | 0.06 | 0.06 | 0.06 | 0.13 | 0.06 | 0.03 | 0.06 | 0.03 | 0.06 | 0.03 |
| Early_inf | class.error | 1.00 | 1.00 | 1.00 | 1.00 | 1.00 | 0.83 | 0.83 | 1.00 | 0.83 | 1.00 | 0.95 | 0.08 |
| Acute_inf | class.error | 0.13 | 0.13 | 0.13 | 0.13 | 0.13 | 0.00 | 0.13 | 0.13 | 0.13 | 0.13 | 0.11 | 0.04 |
| Out-of-bag | OOB | 0.27 | 0.27 | 0.27 | 0.27 | 0.27 | 0.23 | 0.23 | 0.27 | 0.23 | 0.27 | 0.26 | 0.02 |

SD: standard deviation; OOB,:out-of-bag error
